# Prevalence of and factors related to mild and substantial dizziness in community-dwelling older adults: a cross-sectional study

**DOI:** 10.1186/s12877-016-0335-x

**Published:** 2016-09-02

**Authors:** Ann-Sofi C. Kammerlind, Marie Ernsth Bravell, Eleonor I. Fransson

**Affiliations:** 1 Futurum, Region Jönköping County, SE-551 85 Jönköping, Sweden; 2 Department of Medical and Health Sciences, Linköping University, Linköping, Sweden; 3 Institute of Gerontology, School of Health and Welfare, Jönköping University, Jönköping, Sweden; 4 Department of Natural Science and Biomedicine, School of Health and Welfare, Jönköping University, Jönköping, Sweden; 5 Institute of Environmental Medicine, Karolinska Institutet, Stockholm, Sweden

**Keywords:** Dizziness, Older persons, Diseases, Drugs, Blood pressure, Physical activity, Falling, Fear of falling, Quality of life, Physical performance

## Abstract

**Background:**

Dizziness is highly prevalent among older people and associated with many health factors. The aim of the study was to determine the prevalence of and factors related to dizziness among community-dwelling older adults in Sweden. In contrast to previous studies, the subjects with dizziness were divided into two groups, mild and substantial dizziness, according to the frequency and intensity of dizziness.

**Methods:**

A sample of 305 older persons between 75 and 90 years of age (mean age 81 years) were interviewed and examined. Subjects with dizziness answered the University of California Los Angeles Dizziness Questionnaire and questions about provoking movements. The groups with substantial, mild, or no dizziness were compared with regard to age, sex, diseases, drugs, blood pressure, physical activity, exercises, falls, fear of falling, quality of life, general health, mobility aids, and physical performance.

**Results:**

In this sample, 79 subjects experienced substantial and 46 mild dizziness. Subjects with substantial dizziness were less physically active, reported more fear of falling, falls, depression/anxiety, diabetes, stroke/TIA, heart disease, a higher total number of drugs and antihypertensive drugs, lower quality of life and general health, and performed worse physically.

**Conclusions:**

There are many and complex associations between dizziness and factors like falls, diseases, drugs, physical performance, and activity. For most of these factors, the associations are stronger in subjects with substantial dizziness compared with subjects with mild or no dizziness; therefore, it is relevant to differ between mild and substantial dizziness symptoms in research and clinical practice in the future.

## Background

Dizziness, defined as false perceptions of movement or spatial orientation, is highly prevalent in older people [1], related to lower health-related quality of life [2], and a strong contributor to disability [3]. Dizziness can be caused by dysfunctions of the balance system, either in the sensory parts (visual, vestibular, or somatosensory systems) and/or the central nervous system. When the function is significantly impaired in at least two of the three main sensory parts of the balance system, it is called multisensory dizziness [4]. Also, factors external to the balance system such, as medications [5, 6] and cardiovascular diseases [7], can lead to dizziness. Dizziness in older adults is suggested to be a multifactorial geriatric syndrome [7], but may also have single causes, including benign paroxysmal positional vertigo (BPPV) or vestibular impairment [8]. Although BPPV is often unrecognized in the elderly, Kollén et al. [9] found the prevalence to be 11 % in a population of 75-year-olds. Particle repositioning manoeuvres can effectively reduce both dizziness, and as a preliminary finding falls, in elderly people with BPPV [10]. Unilateral vestibular dysfunction results in both dizziness and impaired balance and is more prevalent in fallers versus non-fallers in community-dwelling older adults [11], as well as in elderly subjects with multisensory dizziness [12]. Vestibular rehabilitation interventions [13] are effective in unilateral vestibular function disorders [14] as well as in many other diagnoses of dizziness [13].

Associations between dizziness and some other factors like falls, fear of falling, diseases, and drugs have been found in previous studies [1, 7, 15–18]. In most previous research about the prevalence of dizziness and how it relates to other health factors, dizziness has been studied as a dichotomous variable, dizzy or not. The aim of the study was to determine the prevalence of and factors related to dizziness among community-dwelling older adults in Sweden. In contrast to previous studies, the subjects with dizziness were divided into two groups, mild and substantial dizziness, according to the frequency and intensity of dizziness.

## Methods

### Study design and participants

In this cross-sectional study the data were derived from a population-based study of health, functioning, and mobility among older persons in Sweden [19]. The study population was randomly selected from a population register. Men and women, 75-, 80-, 85-, or 90 years-old, living in Jönköping County, Sweden in 2009 and 2010, were eligible. Persons with dementia were excluded. In total, 327 persons participated in this study, which included a home visit with interviews and examinations performed by trained nurses in the homes of the participants. The interviews were based on a large number of questions and questionnaires regarding, for example, health, quality of life, social networks, activities of daily living (ADL), and physical activity. The examinations included tests of cognitive function, blood pressure, and physical performance. The present study excluded subjects with no or incomplete results for the questions about having dizziness or not (*n* = 12) and subjects living in nursing homes (*n* = 10), yielding an analytical sample of 305 participants (Table 1).

**Table 1:** Background characteristics of study participants and the prevalence of dizziness (*n* = 305)

Sex, *n* (%)	
Females	159 (52)
Males	146 (48)
Age, years, mean (SD)	81 (5)
Marital status, *n* (%)	
Married	165 (54)
Widows/widowers	112 (37)
Never been married	13 (4)
Divorced	15 (5)
Living alone, *n* (%)	145 (48)
Type of housing^a^, *n* (%)	
Living in their own house	157 (51)
Living in an apartment	148 (49)
Dizziness, *n* (%)	
No dizziness	180 (59)
Mild dizziness	46 (15)
Substantial dizziness	79 (26)

^a^The present study excluded subjects living in nursing homes

### Dizziness measures

Subjects were asked if they experienced dizziness (yes/no), and if so, if the dizziness was provoked by (1) getting up from lying to sitting or from sitting to standing, (2) lying down or turning in bed, or (3) standing or walking. Subjects that perceived dizziness also answered the University of California Los Angeles Dizziness Questionnaire (UCLA-DQ) [20] with five questions about the frequency, intensity, effect on daily activities, impact on quality of life, and fear of dizziness. There were five response options for each item giving 1 (least severe) to 5 (most severe) points each, and a total score ranging from 5 to 25. The Swedish version of the scale has good test-retest reliability in patients with dizziness and/or disequilibrium after acute unilateral vestibular loss (ICC 0.89) and central neurological dysfunction (ICC 0.82) [21]. Subjects who reported the least severe response choice for the items of frequency (rarely) and/or intensity (very mild) were categorized as having mild dizziness, and subjects reporting more severe symptoms were categorized as having substantial dizziness [22].

### Measures of potential factors influencing dizziness

Information on age and sex was available. Subjects were asked to list all current diseases and drugs and, based on this information, we derived information about self-reported diabetes, hypertension, stroke/transient ischemic attack (TIA), and heart disease (for example heart failure, atrial fibrillation, and previous myocardial infarction) as well as use of antihypertensive drugs, drugs used in diabetes, and benzodiazepines.

Blood pressure was measured twice when sitting (after at least 5 min rest in sitting) and twice in standing position (immediately and 1 min after standing up) and the averages of the two values in each position were used. Orthostatic hypotension was defined as a systolic blood pressure decrease of at least 20 mmHg and/or a diastolic blood pressure decrease of at least 10 mmHg within 1 min when standing up compared with sitting [23]. Hypertension was defined as a systolic blood pressure of more than 140 mmHg and/or a diastolic blood pressure of more than 90 mmHg in sitting position [24].

Physical activity during the last summer and winter seasons was assessed on a scale ranging from hardly any physical activity to hard training several times per week. The subjects were then categorized as physically active (at least light physical exertion 2-4 h per week) or not (mostly sitting or hardly no physical activity). Subjects were also asked if they performed exercises to maintain or increase joint mobility, strength, or balance on a scale from daily to never. The subjects were then categorized as performing exercises regularly (at least several times per week) or not (never or less than several times per week).

### Measures of potential consequences of dizziness

Subjects were asked if they were afraid of falling (not at all, slightly, quite, or very afraid) and if they had fallen during the last year (not at all, occasionally: 1–2 times, or repeatedly: ≥3 times). The 9-item short version of the Center for Epidemiologic Studies Depression Scale (CESD-9) [25] was used as a measure of depression/anxiety [26]. The questionnaire consists of nine questions summarized into a total score (range 0–9). Quality of life was scored on a scale from 1 to 7, with a score of 1 representing very good and a score of 7 representing very bad quality of life. General health was assessed on a scale from 1 to 5, with a score of 1 representing excellent and a score of 5 representing poor general health. Subjects were also asked to list current mobility aids.

The Short Physical Performance Battery (SPPB) was used for assessment of physical performance. The battery consists of three timed tests: standing balance, walking 3 m, and five chair-stands. Timed results from each test are rescored from 0 (worst performance) to 4 (best performance) and summarized into a total score (range 0–12) [27]. The SPPB is valid and reliable (ICC 0.83–0.89) in adults aged 65–74 years, without severe difficulties with ADL [28].

### Statistical analysis

The three groups with substantial, mild, or no dizziness were compared using one-way Anova (post-hoc Bonferroni) for scale data, Kruskal-Wallis test (post-hoc Dunn’s test) for ordinal data with at least five categories, and chi-squared test (also for post-hoc) for nominal data or ordinal data with four or less categories. To further evaluate the potential factors influencing dizziness, a multivariable multinomial logistic regression was performed with dizziness as the outcome variable. The factors that were statistically significant in the bivariate analyses were considered to be included in the multivariable model. To avoid multicollinearity between strongly related factors, such as diabetes and the use of drugs for diabetes, only one of the variables were included in the model (in this case diabetes). Furthermore, physical activity during summer and winter were collapsed into one variable. Analyses were performed using SPSS 20.0. Statistical significance was defined as *p* ≤ 0.05.

## Results

The present study included a total of 305 community-dwelling elderly subjects with a mean age of 81 (range 75–90) years. Some background characteristics of the subjects are presented in Table 1.

### Prevalence of dizziness

Of the 305 subjects, 125 (41 %) experienced dizziness; of the subjects with dizziness, 79 were categorized as having substantial dizziness and 46 as having mild dizziness according to the UCLA-DQ. For the 125 subjects reporting dizziness, the median (q1-q3) value was 9 (7–13) points on the UCLA-DQ. Dizziness when getting up from lying to sitting or from sitting to standing were the most common movements provoking dizziness, followed by dizziness when standing or walking and dizziness when lying down or turning in bed. A majority (65 %) of the subjects with mild dizziness reported only one of these three dizziness types based on provoking movements. Two or three dizziness types were more commonly reported in subjects with substantial dizziness. The dizziness symptoms for the two groups with mild and substantial dizziness are described in Table 2.

**Table 2:** Description of dizziness symptoms in subjects with mild or substantial dizziness (*n* = 125)

	Mild dizziness (*n* = 46)	Substantial dizziness (*n* = 79)
UCLA Dizziness Questionnaire, (median (Q1-Q3))		
Frequency (1 = rarely, 5 = always)	1 (1–1)	2 (2–4)
Intensity (1 = very mild, 5 = severe)	2 (1–2)	3 (2–3)
Effect on daily activities (1 = no effect, 5 = unable to continue any activities)	1 (1–2)	2 (1–3)
Impact on quality of life (1 = no impact, 5 = severe impact)	1 (1–1)	2 (1–3)
Fear of dizziness (1 = never worry, 5 = always worry)	1 (1–2)	1 (1–3)
Total score (range 5–25)	6 (6–8)	12 (9–15)
Dizziness types based on movements provoking dizziness, *n* (%)^a^		
Getting up from lying to sitting or from sitting to standing	34 (74)	66 (84)
Lying down or turning in bed	7 (15)	23 (29)
Dizziness when standing or walking	25 (54)	58 (73)
Number of dizziness types based on provoking movements, *n* (%)		
One type of dizziness	30 (65)	28 (36)
Two types of dizziness	12 (26)	31 (40)
Three types of dizziness	4 (9)	19 (24)

^a^Subjects may have reported several dizziness types

### Potential factors influencing dizziness

Neither age nor sex differed between the groups with no, mild, or substantial dizziness. Diabetes, stroke/TIA, and heart disease were more common in subjects with substantial dizziness, but self-reported hypertension did not differ between groups. The total number of drugs and the total number of antihypertensive drugs were significantly higher in subjects with substantial dizziness. For the subgroups of antihypertensive drugs, only diuretics were significantly more common in subjects with substantial dizziness, as shown in Table 3.

**Table 3:** Potential factors influencing dizziness compared between the three groups with no, mild, or substantial dizziness (*n* = 305)

	No dizziness (*n* = 180)	Mild dizziness (*n* = 46)	Substantial dizziness (*n* = 79)	*p*-value	Post-hoc
Age, years (mean (SD))	80 (5)	81 (5)	81 (5)	0.081^b^	
Sex, female, *n* (%)	86 (48)	27 (59)	46 (58)	0.188^c^	
Diseases listed by the subjects					
Diabetes, *n* (%)	12 (7)	2 (4)	15 (19)	**0.003** ^c^	S > M, S > N^c^
Hypertension, *n* (%)	63 (35)	19 (41)	26 (33)	0.629^c^	-
Stroke, TIA, *n* (%)	4 (2)	2 (4)	8 (10)	**0.020** ^c^	S > N^c^
Heart disease, *n* (%)	35 (19)	9 (20)	30 (38)	**0.004** ^c^	S > M, S > N^c^
Drugs, n (mean (SD)) at least several times a week	3.5 (2.9)	3.7 (2.6)	5.7 (3.4)	**<﻿﻿0.001** ^b^	S > M, S > N^d^
ACE inhibitors (ATC code C09A^a^), *n* (%)	40 (22)	9 (20)	27 (34)	0.081^c^	
Beta blocking agents (ATC code C07), *n* (%)	63 (35)	17 (37)	35 (44)	0.361^c^	
Diuretics (ATC code C03), *N* (%)	53 (29)	12 (26)	37 (47)	**0.012** ^c^	S > M, S > N^c^
Calcium channel blockers (ATC code C08), *n* (%)	26 (14)	8 (17)	13 (16)	0.846^c^	
All antihypertensive drugs (ATC codes C02, C03, C07, C08, C09A, C09B), *n* (%)	108 (60)	25 (54)	60 (76)	**0.020** ^c^	S > M, S > N^c^
Drugs used in diabetes (ATC code A10), *n* (%)	12 (7)	2 (4)	13 (16)	**0.019** ^c^	S > M, S > N^c^
Blood pressure, measured					
Systolic blood pressure, mmHg (mean (SD))	148 (21)	149 (21)	147 (22)	0.809^b^	-
Diastolic blood pressure, mmHg (mean (SD))	77 (12)	77 (12)	75 (11)	0.285^b^	-
Orthostatic hypotension, *n* (%)	16 (9)	11 (24)	9 (11)	**0.021** ^c^	M > N^c^
Hypertension, *n* (%)	100 (56)	29 (63)	45 (57)	0.558^c^	-
Physical activity and exercises					
Physically active last summer, *n* (%)	157 (87)	40 (87)	56 (71)	**0.004** ^c^	S < M, S < N^c^
Physically active last winter, *n* (%)	141 (78)	37 (80)	45 (57)	**0.001** ^c^	S < M, S < N^c^
Performs joint mobility exercises, *n* (%)	65 (36)	21 (46)	32 (41)	0.460^c^	-
Performs strength exercises, *n* (%)	44 (24)	9 (20)	17 (22)	0.735^c^	-
Performs balance exercises, *n* (%)	30 (17)	10 (22)	13 (16)	0.698^c^	-

*N* no dizziness group, *M* mild dizziness group, *S* substantial dizziness group

Numbers in bold type = p=<0.05

^a^No subjects reported drugs with ATC-code C09B

^b^One-way Anova

^c^Chi-squared test

^d^Bonferroni

Neither systolic or diastolic blood pressure nor the proportion of subjects classified as having hypertension by measured blood pressure, differed between groups. However, the proportion of subjects with orthostatic hypotension was significantly higher in subjects with mild dizziness compared with subjects without dizziness. Fewer subjects with substantial dizziness had been physically active last summer and winter, but the proportion of subjects regularly performing specific mobility, strength, or balance exercises was not significantly different between groups, as shown in Table 3.

In the multivariable multinomial logistic regression including the factors diabetes, stroke/TIA, heart disease, total number of drugs, diuretics, orthostatic hypotension and physical activity, only orthostatic hypotension was statistically significant in relation to mild dizziness [odds ratio (OR) 3.43, 95 % confidence interval (CI) 1.41–8.35, *p* = 0.007] (Table 4). Total number of drugs [OR 1.12, 95 % CI 1.01–1.26, *p* = 0.040), stroke [OR 4.14, 95 % CI 1.13–15.09, *p* = 0.032] and physical inactivity [OR 2.16, 95 % CI 1.01–4.61, *p* = 0.046] remained statistically significant in the association with substantial dizziness in the multivariable model (Table 4).

**Table 4:** Potential factors influencing dizziness evaluated by multivariable multinomial logistic regression, no dizziness used as reference category

	OR^a^	95 % CI^b^	*p*-value
Mild dizziness			
Diabetes (ref. no diabetes)	0.64	0.13–3.24	0.589
Stroke, TIA (ref. no stroke, TIA)	1.81	0.30–10.87	0.515
Heart disease (ref. no heart disease)	0.99	0.40–2.46	0.978
Number of drugs (cont.)	1.04	0.90–1.20	0.570
Diuretics (ref. no diuretics)	0.84	0.37–1.91	0.679
Orthostatic hypotension (ref. no orthostatic hypotension)	3.43	1.41–8.35	**0.007**
Physical inactivity both summer and winter (ref. physical activity both summer and winter)	0.75	0.26–2.16	0.588
Physical inactivity winter, physical activity summer (ref. physical activity both summer and winter)	0.41	0.09–1.93	0.257
Substantial dizziness			
Diabetes (ref. no diabetes)	1.57	0.63–3.93	0.335
Stroke, TIA (ref. no stroke, TIA)	4.14	1.13–15.09	**0.032**
Heart disease (ref. no heart disease)	1.67	0.86–3.27	0.133
Number of drugs (cont.)	1.12	1.01–1.26	**0.040**
Diuretics (ref. no diuretics)	1.21	0.64–2.28	0.555
Orthostatic hypotension (ref. no orthostatic hypotension)	0.99	0.38–2.58	0.990
Physical inactivity both summer and winter (ref. physical activity both summer and winter)	2.16	1.01–4.61	**0.046**
Physical inactivity winter, physical activity summer (ref. physical activity both summer and winter)	1.94	0.79–4.76	0.147

Numbers in bold type = p=<0.05

^a^OR = odds ratio

^b^95 % CI = 95 % confidence interval

### Potential consequences of dizziness

Subjects with substantial dizziness reported both a higher degree of fear of falling and more falls during the previous year. They also rated higher levels of depression/anxiety, lower quality of life, and lower general health. More subjects with substantial dizziness used mobility aids and they showed a worse physical performance measured with the SPPB, as shown in Table 5. The same pattern of significant differences between the three groups was seen for all three sub scores (balance, walking, and chair-stands) of the SPPB (data not shown).

**Table 5:** Potential consequences of dizziness compared between the three groups with no, mild, or substantial dizziness (*n* = 305)

	No dizziness (*n* = 180)	Mild dizziness (*n* = 46)	Substantial dizziness (*n* = 79)	*p*-value	Post-hoc
Fear of falling				<**0.001** ^b^	S > M, S > N^b^
Not afraid at all, *n* (%)	101 (56)	30 (65)	29 (37)		
Slightly afraid, *n* (%)	61 (34)	11 (24)	24 (30)		
Quite afraid, *n* (%)	15 (2)	3 (7)	18 (23)		
Very afraid, *n* (%)	3 (2)	2 (4)	8 (10)		
Falling last year				**0.001** ^b^	S > N^b^
Not at all, *n* (%)	128 (72)	28 (61)	36 (46)		
Occasionally (1–2 times), *n* (%)	46 (26)	14 (30)	36 (46)		
Repeatedly (≤3 times), *n* (%)	4 (2)	4 (9)	7 (9)		
Depression/anxiety					
CESD-9 total score (median (Q1-3))	0 (0–2)	1 (0–2)	1 (0–3)	**0.005** ^c^	S > N^d^
Quality of life^a^ (median (Q1-3))	2 (1–3)	2 (1–2)	2 (2–3)	0.002^c^	S > M, S > N^d^
General health^a^ (median (Q1-3))	3 (2–3)	3 (3–4)	3 (3–4)	<**0.001** ^c^	S > N^d^
Using one or several mobility aids, *n* (%)	38 (21)	12 (27)	47 (60)	<**0.001** ^b^	S > M, S > N^b^
Physical performance					
SPPB total score (median (Q1-3))	10 (8–12)	10 (8–11)	7 (5–9)	<**0.001** ^c^	S < M, S < N^d^

*N* = No dizziness group; *M* = Mild dizziness group; *S* = Substantial dizziness group; *SPPB* = Short Physical Performance Battery

Numbers in bold type = p=<0.05

^a^A lower value means better quality of life/general health

^b^Chi-squared test

^c^Kruskal-Wallis test

^d^Dunns test

## Discussion

In this study, 41 % of community-dwelling older adults reported dizziness, most commonly when getting up from lying to sitting or from sitting to standing indicating a drop in blood pressure. Several associations were found between dizziness and other health factors. In most previous research in this area, dizziness has been studied as a dichotomous variable, either dizziness or not, or persistent/troublesome dizziness or not [1, 16, 29]. In the present study, we divided the subjects into three groups: no, mild, or substantial dizziness. The comparisons, with a few exceptions, showed differences between the group with substantial dizziness compared with the mild group and the group without dizziness. The few differences between subjects with mild and no dizziness indicate it is relevant to differentiate between mild and substantial dizziness symptoms.

The 41 % prevalence of dizziness among community-dwelling elderly subjects with a mean age of 81 years was in line with previous research [7, 17]. Dizziness when getting up as the most common movement provoking dizziness followed by dizziness when standing or walking confirms the findings in a previous Swedish population study [30]. Thirty subjects (10 %) reported dizziness when lying down or turning in bed which may likely be caused by BPPV, previously found to be present in 9-11 % of elderly populations [9, 31].

Age, sex, diseases, drugs, blood pressure, physical activity, and different exercises were the potential factors influencing dizziness measured in this study. In some other studies, dizziness has been more clearly associated with age compared with our study, and this may be explained by a larger age range in those studies [7, 17]. Probably it is mainly other age-related factors like diseases and drugs, rather than normal aging that explain the relationship between age and dizziness [3]. Stevens et al. [1] performed multivariate analyses and did not find dizziness, but balance problems, to be associated with age.

The association found between dizziness and diabetes may be explained by complications from the disease in different components of the balance system. For example, glucose metabolism has a significant impact on the physiology of the inner ear, and 60 % of the patients with diabetes type 1 or 2 showed vestibular system changes in a study by Klagenberg et al. [32]. In addition, drugs used in diabetes, like insulin and analogues, were associated with dizziness but this may also be explained by the association between diabetes and dizziness. Some earlier studies have shown a higher frequency of dizziness among subjects with diabetes compared with healthy controls [7, 33], but others have not [1]. There was a higher prevalence of self-reported previous stroke or TIA in the group with substantial dizziness. Colledge et al. [15] and Stevens et al. [1] did not find an association between dizziness and either diabetes or stroke, which may be due to a different selection of subjects and different definition of dizziness.

There was no difference between the groups regarding self-reported hypertension, and this is in line with previous findings by Stevens et al. [1]. In our study, fewer subjects (*n* = 108) reported hypertension compared with the number of subjects with measured hypertension (*n* = 174) and the number of subjects using antihypertensive drugs (*n* = 193). Subjects may not be aware of high blood pressure, and they may not report having hypertension if they use antihypertensive drugs; thus, self-reporting may be a less reliable measure of hypertension. More subjects reported dizziness when getting up from lying to sitting or from sitting to standing (*n* = 100) compared with the number of subjects having measured orthostatic hypotension (*n* = 36). Possible explanations may be that blood pressure falls that are less dramatic than those defined as orthostatic hypotension can give symptoms, and also that some subjects may have a progressive decrease of blood pressure over time frames as long as 10–15 min [34]. In contrast to most of the other variables, orthostatic hypotension was significantly more prevalent only in those with mild dizziness compared with subjects with no dizziness. This may be a chance finding, or maybe orthostatic hypotension causes mainly mild dizziness. Measured systolic or diastolic blood pressure, or hypertension defined by measured blood pressure, did not differ between subjects with or without dizziness. In a previous study by Abate et al. [35], elderly community-dwelling and otherwise healthy subjects with hypertensive blood pressure complained more frequently about dizziness compared with normotensive subjects. However, in that study [35], however, not only subjects fulfilling the criteria of a systolic blood pressure of more than 140 mmHg and/or and diastolic blood pressure of more than 90 mmHg were categorized as hypertensive, but also subjects with a history of hypertension and subjects using antihypertensive drugs. In line with this, the use of antihypertensive drugs, and diuretics in particular, was associated with dizziness in the present study, as in previous studies [36, 37]. Antihypertensives may cause generalized or orthostatic hypotension and thereby dizziness [6]. The use of antihypertensives is also associated with falls [38], especially during the first 14 days after drug initiation [39]. Heart disease was also more prevalent in the group with substantial dizziness in the present study. Associations have previously been found between dizziness and abnormal heart rhythm [1], angina [15], and previous myocardial infarction [15].

The association between dizziness and the number of medications is probably explained by both the medications (side effects) and the underlying diseases. Gassman et al. [7] found that daily dizziness and a longer duration of dizziness were associated with daily intake of four or more drugs. Polypharmacy is also associated with a greater fall risk, but recent studies have shown that this association is only true if medications that increase the risk of falling are included [40]. Also, benzodiazepines (ATC codes N05BA, N05CD) are known to correlate with dizziness [6], but those drugs were not included in the present analyses since they were reported by very few subjects.

Fewer subjects with substantial dizziness were physically active in the past summer and winter. Gassman et al. [7] found that daily dizziness was associated with an inactive lifestyle. Inactivity may increase the risk of dizziness and impaired balance [41], but dizziness and balance problems may also prevent people from exercising and being physically active [42]. No differences were seen between groups, regarding regularly performed joint mobility, strength, or balance exercises. Being generally more physically active, and/or performing more dizziness-specific exercises, such as those used in vestibular rehabilitation [13], may be more important than joint mobility, strength, or balance exercises to counteract dizziness.

Fear of falling, a history of falling, quality of life, general health, use of mobility aids, and physical performance were the potential consequences of dizziness measured in this study. Dizziness has previously been found to be associated with falls [1, 7, 16, 17, 43], an increased injury rate from falls [43], and the fear of falling [7, 16, 18]. Both falls and dizziness in the elderly often have multifactorial aetiologies, and many of the risk factors are the same for both these conditions [7]. These associations between dizziness, falls, and fear of falling were confirmed in the present study and there are several possible causal relationships. Other factors like diseases and dysfunctions of the balance systems may be the cause of all three factors: dizziness, falls, and fear of falling. It is also possible that dizziness is a cause of falling and fear of falling, and/or that falling and fear of falling have led to inactivity, causing dizziness because of inadequate stimulation of the balance system.

CESD-9 was used as a measure of depression and anxiety. The long version of the instrument CES-D has been showed to be equally associated with current depression as well as current generalized anxiety [26]. Dizzy subjects are more likely to report depression and anxiety as shown in this as well as previous [7, 17, 44] studies. Anxiety can cause dizziness, and dizziness ca trigger both anxiety and depression [45]. Menant et al. [44] found anxiety to be a mediator between dizziness and falls in older community-dwelling adults, suggesting that older dizzy people may fall because of factors related to anxiety.

In line with previous studies [2, 7], dizziness was related to lower quality of life and lower perceived general health. Furthermore, inactivity may cause both dizziness and a low quality of life. Ekwall et al. [41] showed that physical activity correlated with a reduced risk of low quality of life in elderly subjects with dizziness. Subjects with substantial dizziness used mobility aids to a higher degree and showed a lower physical performance, as measured by the SPPB, in our study. Impaired function of the balance system might have caused both the dizziness and the impaired physical performance. It is also likely that dizziness has led to avoidance of movements and activities, which in turn have worsened the dizziness and caused balance problems and reduced strength. Gassman et al. [7] found daily dizziness to be associated with difficulties while walking 500 m or getting up from bed, and a longer duration of dizziness was associated with the use of walking aids. Stevens et al. found an association between dizziness and both the SPPB and a reduced grip strength.

The strengths of the present study are the representative sample of 305 community-dwelling older persons, the categorization into mild or substantial dizziness, and the many measures covering a wide range of health related factors. One limitation is that no otoneurological examinations were done to determine the causes of dizziness in each subject. We did not use an accepted measure for anxiety, but for depression.

Since dizziness impairs both physical and mental health related quality of life, it is important to focus on both clinical symptoms and psychosocial factors in the care of subjects with dizziness [2]. Elderly dizzy subjects should be thoroughly assessed, and the cause of dizziness should be determined to enable targeted and effective treatments.

## Conclusions

Dizziness is highly prevalent among older people. There are many and complex associations between dizziness and factors like falls, diseases, drugs, physical performance, and activity. For most of these factors, the associations are stronger in subjects with substantial dizziness compared with subjects with mild or no dizziness; therefore, it is relevant to differ between mild and substantial dizziness symptoms in research and clinical practice in the future. More research is needed to further explore the causality of the relationships, and how to most effectively counteract vicious circles and reduce dizziness in older adults.

## Abbreviations

ADL: Activities of daily living
BPPV: Benign paroxysmal positional vertigo
SPPB: Short physical performance battery
TIA: Transient ischemic attack
UCLA-DQ: University of California Los Angeles Dizziness Questionnaire
